# Disability Status in LGBT Adults by Sex and Age

**DOI:** 10.1001/jamanetworkopen.2025.21454

**Published:** 2025-07-16

**Authors:** Aarya Suryavanshi, Jonathan Cantor, Megan S. Schuler

**Affiliations:** 1 RAND, Santa Monica, California; 2 RAND, Arlington, Virginia

## Abstract

This cross-sectional study compares rates of self-reported disability among individuals who identify as lesbian, gay, bisexual, or transgender (LGBT), stratifying by sex and age.

## Introduction

Growing evidence from US survey data suggests that lesbian, gay, bisexual, transgender, and queer (LGBTQ) individuals report higher rates of disability compared with non-LGBTQ peers. Data from the 2020 Behavioral Risk Factor Surveillance System showed that 36% of LGBTQ respondents reported a disability compared to 24% of non-LGBTQ respondents, and transgender individuals reported higher disability rates than cisgender individuals.^1,2^ Among the general population, women and older adults report higher rates of disability.^3^ This study examines whether sex at birth or age modifies the association of disability with LGBT status.

## Methods

This cross-sectional study was determined exempt from review and the requirement of informed consent by the RAND institutional review board; reporting follows the STROBE reporting guideline. We analyzed data from the Household Pulse Survey (phase 4.1, cycles 4-6), collected by the National Center for Health Statistics and the US Census Bureau between April 2 and June 24, 2024. The survey asked about sexual identity, gender identity, and sex at birth. Following Census guidelines, based on these items, respondents were classified as LGBT if they identified as gay, lesbian, bisexual, transgender, or if their current gender identity differed from their sex at birth; all other respondents were classified as non-LGBT (ie, straight and cisgender) (eMethods in Supplement 1).^4^ Disability was measured with the Washington Group Statistics Short Set. Respondents were classified as having any disability if they reported a lot of difficulty or cannot do at all on any item, per Washington Group guidelines.^5^ Key moderators of interest included sex at birth (male or female) and age group (18-34, 35-59, and ≥60 years). Additional covariates included self-reported Hispanic ethnicity and self-reported race (Asian, Black, White, and multiracial or other race [ie, any race other than Asian, Black, or White]); race and ethnicity were included to control for differences.

We reported sociodemographic characteristics of LGBT and non-LGBT respondents and tested for differences across groups with survey-weighted χ^2^ tests. We reported survey-weighted disability rates, stratified by LGBT identity and age group for males and females. Analyses were conducted in Stata version 18.0 (StataCorp).

## Results

Our sample comprised 130 146 adults, 16 195 who identified as LGBT and 113 951 who identified as non-LGBT (Table). Of the LGBT group, 60.7% (95% CI, 59.5%-62.0%) identified as bisexual, 35.5% (95% CI, 34.2%-36.7%) as gay or lesbian, and 8.6% (95% CI, 7.7%-9.7%) as transgender. LGBT respondents, compared with non-LGBT respondents, were younger (mean age, 37.3 [95% CI, 37.0-37.7] vs 50.3 [95% CI, 50.2-50.5] years) and a greater proportion were female (57.4% [95% CI, 56.0%-59.0%] vs 50.6% [95% CI, 50.2%-51.0%]). LGBT adults were nearly twice as likely to report a disability as non-LGBT adults (18.6% [95% CI, 17.7-19.6%] vs 10.8% [95% CI, 10.5%-11.2%]). There was no significant difference in race and ethnicity distribution by LGBT identity.

**Table.** Disability Status and Demographic Characteristics by LGBT Identity

Characteristic	Participants, No. (weighted %) [95% CI] (N = 130 146)	
	LGBT (n = 16 195)	Non-LGBT (n = 113 951)
Any disability^a^	2395 (18.6) [17.7-19.6]	11 230 (10.8) [10.5-11.2]
Transgender^a^	953 (8.6) [7.7-9.7]	NA
Sexual identity^a^		
Bisexual	8604 (60.7) [59.5-62.0]	NA
Gay or lesbian	7114 (35.5) [34.2-36.7]	NA
Heterosexual	185 (1.0) [0.7-1.3]	113 951 (100)
Something else or do not know	276 (2.8) [2.4-3.4]	NA
Sex at birth^a^		
Male	6946 (42.5) [41.0-44.0]	50 549 (49.4) [49.0-49.8]
Female	9249 (57.5) [56.0-59.0]	63 402 (50.6) [50.2-51.0]
Ethnicity: Hispanic, Latino, or Spanish origin^a^	1726 (19.4) [17.9-20.9]	9493 (16.7) [16.3-17.2]
Race^a^^,^^b^		
Asian, alone	531 (4.2) [3.6-4.9]	5214 (5.6) [5.4-5.9]
Black, alone	1107 (10.6) [9.6-11.8]	9884 (12.9) [12.6-13.2]
White, alone	13 361 (76.2) [74.8-77.6]	93 159 (75.4) [75.0-75.9]
Multiracial or other race	1196 (9.0) [8.2-9.9]	5694 (6.1) [5.8-6.4]
Age, y^a^		
18-34	5244 (55.9) [54.8-57.0]	12 454 (21.6) [21.2-22.0]
35-59	7566 (32.9) [31.9-34.0]	52 543 (44.9) [44.5-45.2]
≥60	3385 (11.2) [10.4-12.0]	48 954 (33.6) [33.2-33.9]
Mean (95% CI)	37.3 (37.0-37.7)	50.4 (50.2-50.5)

Abbreviation: LGBT, lesbian, gay, bisexual, transgender; NA, not applicable.

^a^ Statistically significant difference (at the .05 level) between groups, determined by a weighted χ^2^ test.

^b^ Race data were self-reported and include all categories available in the Household Pulse Survey public use file. Other race includes any race not otherwise specified.

For both sexes, disability disparities between LGBT and non-LGBT adults were most pronounced at younger ages (Figure). Among those aged 18 to 34 years, differences in disability rates for LGBT adults vs non-LGBT adults were statistically significant, with rates for LGBT adults being approximately 3 times higher among females (23.8% [95% CI, 21.6%- 26.2%] vs 7.9% [95% CI, 7.0%-8.8%]) and approximately twice as high among males (14.9% [95% CI, 12.4%-17.7%] vs 8.0% [95% CI, 6.8%-9.4%]). LGBT males and females had significantly elevated disability rates at ages 35 to 59 years. Disability rates increased with age for non-LGBT adults but were relatively flat across age groups for LGBT males and were highest among LGBT females aged 18 to 34 years.

**Figure.**
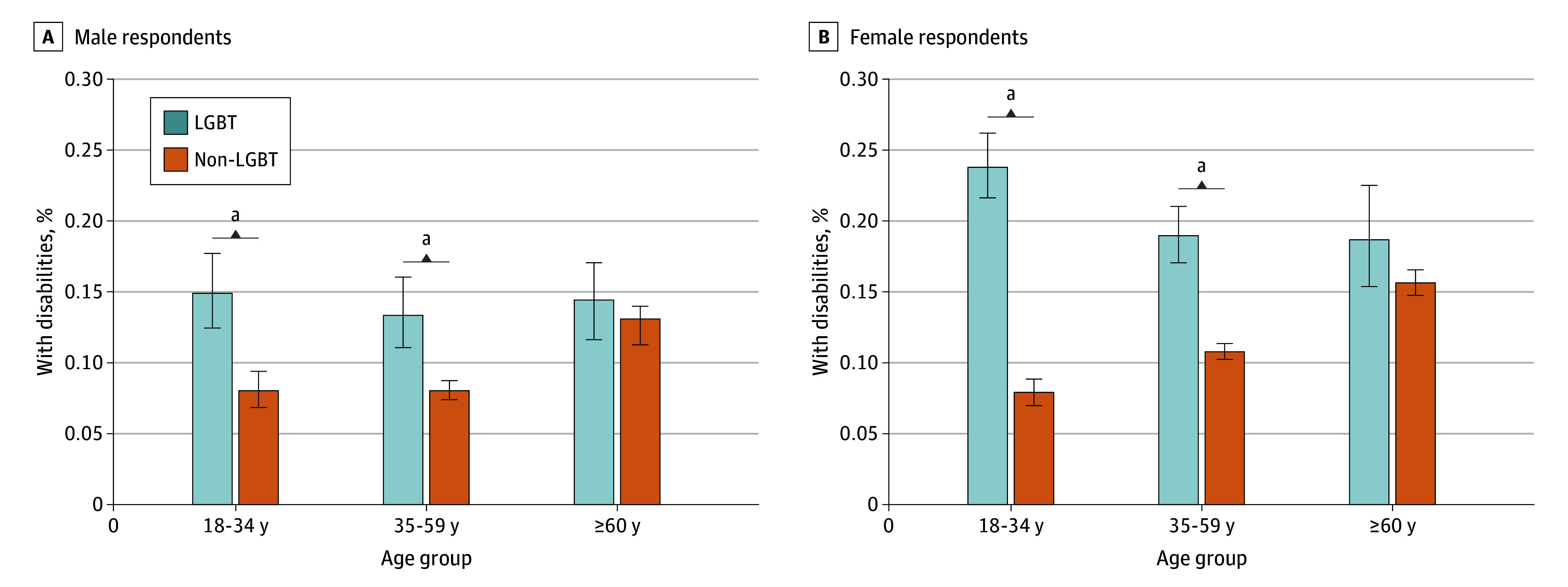
Self-Reported Disability by LGBT Status and Age Group Error bars represent 95% CIs of the percentages. ^a^Statistically significant difference (at .05 level) between LGBT (lesbian, gay, bisexual, transgender) and non-LGBT groups, determined by a weighted χ^2^ test.

## Discussion

This cross-sectional study found that LGBT adults reported disability rates nearly twice as high as non-LGBT adults, with greater disparities among females. Disability rates were highest among LGBT females aged 18 to 34 years, who reported rates 3 times higher than similarly aged non-LGBT females. Minority stress theory might help explain these disparities in disability status due to chronic stigma and discrimination experienced by LGBT individuals. Systemic gender inequality has been theorized to negatively impact women’s health outcomes.^6^ For LGBT women, these effects likely amplify one another, contributing to the sizeable disability disparities observed. Limitations include social desirability and recall biases in responses, the cross-sectional survey design precluding causal inference, and an inability to comprehensively assess some disability types.

## Notes

Supplement 1.
eMethods.

Supplement 2.
Data Sharing Statement
