# Supplementary material for: Disability Status in LGBT Adults by Sex and Age
Source: JAMA Netw Open. 2025 Jul 16;8(7):e2521454. doi: 10.1001/jamanetworkopen.2025.21454 (PMC12268491; doi:10.1001/jamanetworkopen.2025.21454)
Supplement: Supplement 1. — eMethods. [file jamanetwopen-e2521454-s001.pdf]

Supplemental Online Content

Suryavanshi A, Cantor J, Schuler MS. Disability status in LGBT adults by sex and age. *JAMA Netw. Open*. 2025;8(7):e2521454. doi:10.1001/jamanetworkopen.2025.21454

eMethods.

This supplemental material has been provided by the authors to give readers additional information about their work.

© 2025 Suryavanshi A et al. *JAMA Network Open*.

eMethods.

Household Pulse Survey questions about sexual identity, gender identity, and sex at birth were used to classify individuals as LGBT and non-LGBT. In the survey, the sexual orientation item included responses: “Gay or lesbian”, “Straight”, “Bisexual”, “Something else”, and “I don’t know.” The gender identity item included responses: “Male”, “Female”, “Transgender”, and “None of these.” Sex at birth was reported as “Male” or “Female.” Following Census guidelines, respondents were classified as LGBT if: (1) they reported their sexual orientation as “Gay or lesbian” or “Bisexual”, (2) they described their current gender identity as “Transgender”, or (3) their sex at birth is different from their current gender identity.[8] Individuals in the non-LGBT group reported that their sexual orientation was “Straight, that is not gay or lesbian” and their sex at birth aligned with their current gender identity.

© 2025 Suryavanshi A et al. *JAMA Network Open*.
