# Supplementary material for: Disability Status in LGBT Adults by Sex and Age
Source: JAMA Netw Open. 2025 Jul 16;8(7):e2521454. doi: 10.1001/jamanetworkopen.2025.21454 (PMC12268491; doi:10.1001/jamanetworkopen.2025.21454)
Supplement: Supplement 2. — Data Sharing Statement [file jamanetwopen-e2521454-s002.pdf]

Data Sharing Statement

Suryavanshi. Disability Status in LGBT Adults by Sex and Age. *JAMA Netw Open*. Published July 16, 2025. doi:10.1001/jamanetworkopen.2025.21454

Data
Data available: Yes
Data types: Deidentified participant data
How to access data: Data available upon email to the corresponding author.
When available: With publication

Supporting Documents
Document types: Statistical/analytic code
How to access documents: Code available upon email to the corresponding author.
When available: With publication

Additional Information
Who can access the data: Anyone requesting the data.
Types of analyses: For any purpose.
Mechanisms of data availability: Through email with the corresponding author.
